# Video: Clinical evaluation of a laparoscopic hyperspectral imaging system

**DOI:** 10.1007/s00464-022-09282-y

**Published:** 2022-05-11

**Authors:** Annekatrin Pfahl, Hannes Köhler, Madeleine T. Thomaßen, Marianne Maktabi, Albrecht M. Bloße, Matthias Mehdorn, Orestis Lyros, Yusef Moulla, Stefan Niebisch, Boris Jansen-Winkeln, Claire Chalopin, Ines Gockel

**Affiliations:** 1 Innovation Center Computer Assisted Surgery (ICCAS), Faculty of Medicine, Leipzig University, Semmelweisstr. 14, 04103 Leipzig, Germany; 2 Department of Visceral, Transplant, Thoracic, and Vascular Surgery, University Hospital of Leipzig, Leipzig, Germany; 3 Department of General, Visceral, Thoracic, and Vascular Surgery, Klinikum St. Georg, Leipzig, Germany

**Keywords:** Clinical evaluation study, Gastrointestinal surgery, Hyperspectral imaging, Laparoscopic surgery, Minimally invasive surgery

## Abstract

**Background:**

Hyperspectral imaging (HSI) during surgical procedures is a new method for perfusion quantification and tissue discrimination. Its use has been limited to open surgery due to large camera sizes, missing color video, or long acquisition times. A hand-held, laparoscopic hyperspectral camera has been developed now to overcome those disadvantages and evaluated clinically for the first time.

**Methods:**

In a clinical evaluation study, gastrointestinal resectates of ten cancer patients were investigated using the laparoscopic hyperspectral camera. Reference data from corresponding anatomical regions were acquired with a clinically approved HSI system. An image registration process was executed that allowed for pixel-wise comparisons of spectral data and parameter images (StO_2_: oxygen saturation of tissue, NIR PI: near-infrared perfusion index, OHI: organ hemoglobin index, TWI: tissue water index) provided by both camera systems. The mean absolute error (MAE) and root mean square error (RMSE) served for the quantitative evaluations. Spearman’s rank correlation between factors related to the study design like the time of spectral white balancing and MAE, respectively RMSE, was calculated.

**Results:**

The obtained mean MAEs between the TIVITA® Tissue and the laparoscopic hyperspectral system resulted in StO_2_: 11% ± 7%, NIR PI: 14±3, OHI: 14± 5, and TWI: 10 ± 2. The mean RMSE between both systems was 0.1±0.03 from 500 to 750 nm and 0.15 ±0.06 from 750 to 1000 nm. Spearman’s rank correlation coefficients showed no significant correlation between MAE or RMSE and influencing factors related to the study design.

**Conclusion:**

Qualitatively, parameter images of the laparoscopic system corresponded to those of the system for open surgery. Quantitative deviations were attributed to technical differences rather than the study design. Limitations of the presented study are addressed in current large-scale in vivo trials.

**Supplementary Information:**

The online version contains supplementary material available at 10.1007/s00464-022-09282-y.

Since different molecules, e.g. hemoglobin or water, have distinct spectral reflectance properties, this also applies to organs and tissues. By analyzing these spectral signatures, hyperspectral imaging allows conclusions concerning the organ at hand, for example about its oxygenation, water content, or hemoglobin content. These parameters can be visualized in false-color images as shown in Fig. 1

**Fig. 1:**
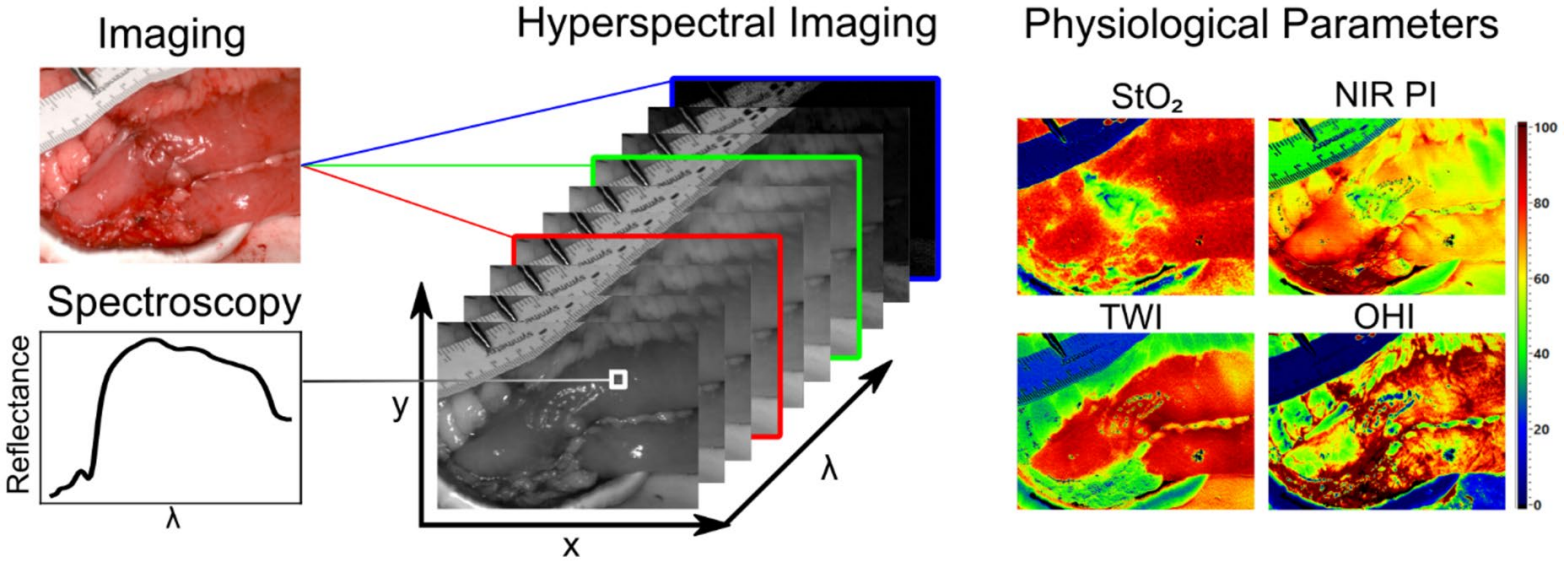
Structure and composition of hyperspectral data and physiological parameters calculated thereof visualized as false-color images. *StO*_2_ oxygen saturation of tissue, *NIR PI* near-infrared perfusion index, *TWI* tissue water index, *OHI* organ hemoglobin index (Color figure online)

Over the last years, hyperspectral imaging (HSI) has been evaluated in different fields of surgery and three promising applications have been discovered: Perfusion measurements [1–6], intraoperative discrimination of risk structures, and tissue differentiation [7–16], especially to distinguish healthy from cancerous tissue. An overview of its capabilities for intraoperative use was given in [17]. Clinical implementation, however, has been limited to open surgery [18].

In robotic and laparoscopic operations, the surgeon’s haptic feedback is reduced. This deficit needs to be compensated by enhanced visualization, as compared to open surgery. HSI could provide the surgeon with additional and more objective information. Although several minimally invasive/robotic HSI cameras are currently being developed, none has made it to routine clinical usage [19–24]. Limitations have been long acquisition times, missing color video, and large set-ups due to camera size. These limitations have been overcome in the new hyperspectral camera for minimally invasive surgery (HSI MIS). Before implementation during surgery, this system was evaluated *ex vivo* in cancer resectates and compared with the open HSI camera.

## Materials and methods

### Study information

This trial was conducted as a non-inferiority study at the Department of Visceral, Transplant, Thoracic, and Vascular Surgery of the University Hospital of Leipzig. Between January and April 2021, ten patients undergoing oncological resections were enclosed, including rectum resections (*n *= 3), colonic resections (*n* = 2), esophagectomies (*n* = 4) and gastrectomies (*n* = 1).

The study was approved by the local ethics committee of the Faculty of Medicine of the Leipzig University (No. 393/16-ek, amendment 1) on 01/30/2020 and registered at clinicaltrials.gov (NCT04230603) on 01/13/2020. All methods were performed according to the declaration of Helsinki and all patients gave written informed consent.

### Data acquisition systems

**Fig. 2:**
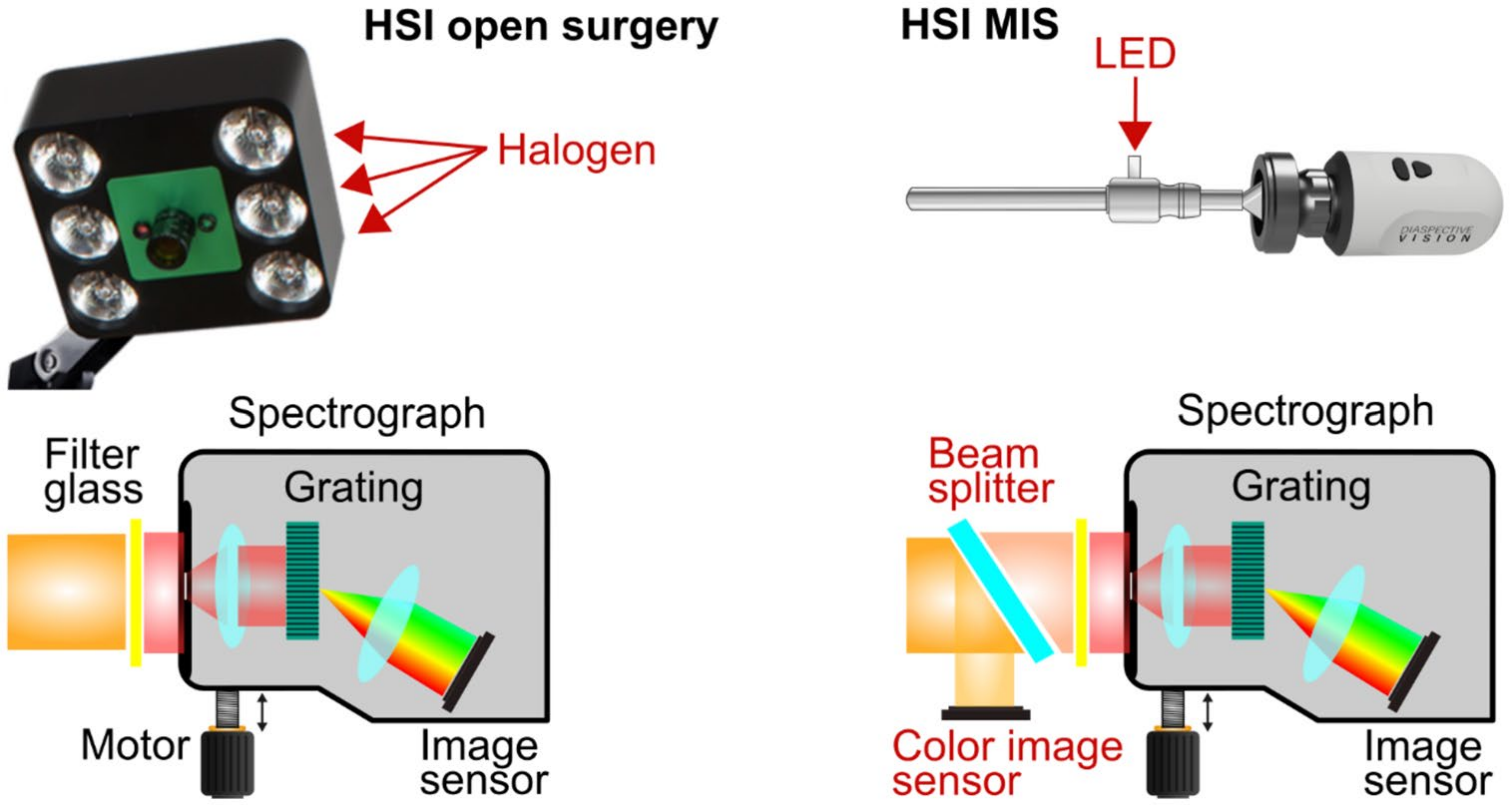
Technical comparison of the HSI systems for open (left) and minimally invasive surgery (right). Differences are highlighted in red. In the upper part, the relevant parts of both systems are shown at a glance beside the corresponding LED light source. Below, the structures of the hyperspectral cameras are illustrated schematically. The HSI MIS contains an additional beam splitter and a color image sensor to provide an RGB video in real-time (Color figure online)

For *ex vivo* data acquisition, the hyperspectral imaging systems TIVITA® Tissue and HSI MIS (both Diaspective Vision GmbH, Am Salzhaff-Pepelow, Germany) were used. Both systems are push-broom scanning systems, measuring spectral information of underlying tissue or organs between 500 and 1000 nm with a spectral resolution of 5 nm. Besides false-color images, representing physiological information on the tissues oxygenation, perfusion, water, and hemoglobin content [25, 26], the TIVITA® Tissue provides a red, green, blue (RGB) color image, whereas a white light video is available in real-time in case of the HSI MIS. There are further technical differences between the light sources and image sensors, illustrated in Fig. 2. Six halogen spots serve for the illumination in the case of the TIVITA® Tissue. Light-emitting diodes (LEDs) provide homogenous lighting for the HSI MIS. The TIVITA® Tissue has one monochromatic image sensor, whereas the HSI MIS includes an additional RGB sensor. The clinical applicability of the TIVITA® Tissue during gastrointestinal surgery and its benefit has been shown in several investigations [27–29] but is limited to open surgery. Here, it has been used as a reference system. The HSI MIS was developed previously for minimally invasive surgery and evaluated technically [18].

### Data acquisition process

During gastrointestinal surgery following the standard operating procedures of the University Hospital of Leipzig and maintaining aseptic standards, *ex vivo* contactless hyperspectral imaging was applied on oncologic rectal, colonic, esophageal, and gastric resectates. The resected organs were cut open and cleaned. In a room next to the operating theater, data were acquired with the HSI MIS first, and the TIVITA® Tissue afterward.

Immediately before the first imaging process, white balancing was conducted. All durations between turning on the light source, white balancing, salvage of the resectate, and the first HSI measurement were recorded. For blur-free recordings, the HSI MIS was mounted 3 cm above the resectate in a laparoscope holding arm.

### Data analyses and statistics

The HSI MIS was evaluated by comparing two different HSI records from:

- the same TIVITA® Tissue system (TvsT),
- two TIVITA® Tissue systems (T1vsT2),
- the HSI MIS (LvsL),
- one TIVITA® Tissue system and the HSI MSI (TvsL).

Recorded HSI images, i.e. the oxygen saturation of tissue (StO2), the near-infrared perfusion index (NIR PI), the tissue water index (TWI), and the organ hemoglobin index (OHI), of the same object were aligned by manual annotation of corresponding points and subsequent perspective transformation. This enabled a pixel-wise comparison of raw spectral data, as well as the calculation of difference images. The mean absolute error (MAE) was determined for each difference image. Afterward, MAE mean and standard deviation (SD) of all records were calculated. Analogously, the root mean square error (RMSE) was determined between the spectral data.

Since deviations between the parameter images and spectral data of both HSI systems might have been affected by the data acquisition process or registration errors, Spearman’s rank correlation coefficients and associated *p*-values were calculated between the MAE of parameter images or the RMSE of the reflectance spectra and

- the time difference between the first HSI measurement and switching on the light source,
- the time difference between the first HSI measurement and the resection,
- the time difference between the first HSI measurement and the white balancing,
- the registration error,
- the mean parameter value.

The significance level was set to *p* = 0.05.

## Results

**Fig. 3:**
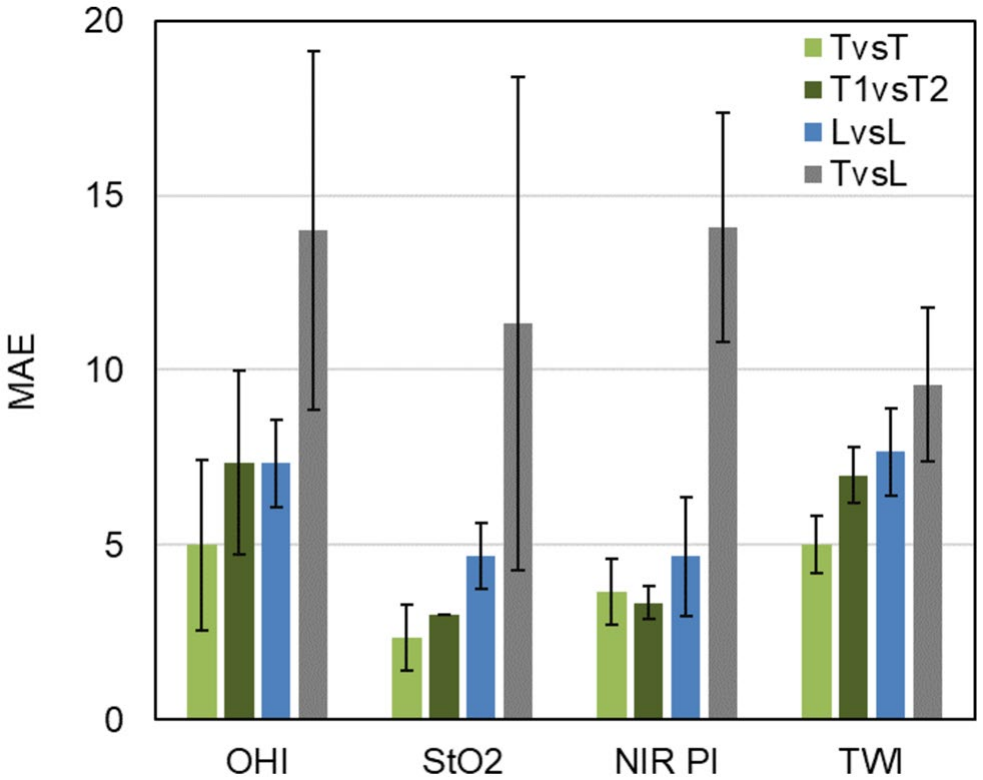
Mean absolute error (MAE) and standard deviation of the calculated tissue parameters for all records from the same (TvsT) and two TIVITA® Tissue systems (T1vsT2), the same HSI MIS system (LvsL), and the difference between both systems (TvsL) (Color figure online)

The image alignment process could be conducted with at least one corresponding dataset from the TIVITA® Tissue systems and the HSI MIS for each investigated resectate. In minute 6:49 of the video file, the alignment result and overlay of both fields of view are shown for one dataset. For two tissue parameters, NIR PI and OHI, the measured values with the HSI MIS were systematically higher and lower, respectively, compared to the TIVITA® Tissue system (TvsL) for all patients. The determined mean MAEs for TvsL were in the same range for all tissue parameters (StO_2_: 11% ±7%, NIR PI: 14 ± 3, OHI: 14± 5, TWI: 10 ± 2). Additionally, the mean MAE for two measurements with the same system and two different TIVITA® Tissue systems (TvsT, LvsL, and T1vsT2) were below 8 units for all tissue parameters as shown in Fig. 3.

**Fig. 4:**
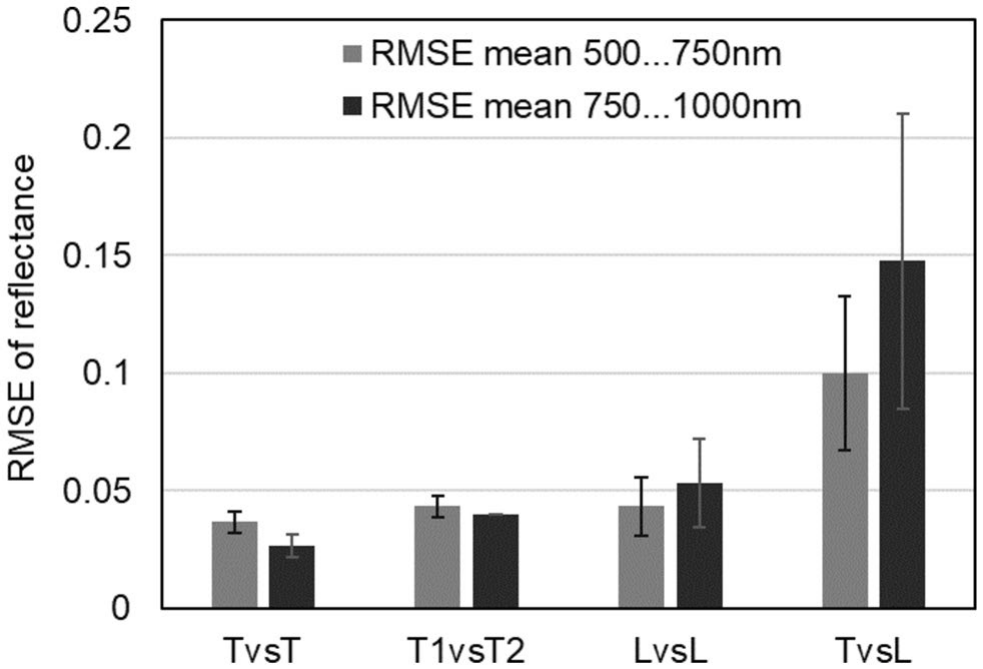
Mean root mean square error (RMSE) and standard deviation for all records in the visible (500 to 750 nm) and near-infrared range (750 to 1000 nm) of the reflectance spectra measured with the same (TvsT) and two TIVITA® Tissue systems (T1vsT2), the same HSI MIS system (LvsL), and both systems (TvsL)

In the visible range (500 to 750 nm), the mean RMSE was similar for TvsT, T1vsT2, and LvsL (≈0.04). Whereas the value decreased in the near-infrared range (750 to 1000 nm) for TvsT and T1vsT2, it increased slightly for LvsL. The mean RMSE between both systems (TvsL) was 0.1 ±0.03 in the visible and 0.15 ± 0.06 in the near-infrared wavelength range as shown in Fig. 4.

The first HSI measurement was acquired 48 min (± 26 min) after switching on the light source, 18 min (±11 min) after spectral white balancing, and 17 min (±7 min) after tissue resection.

The resulting tissue parameter ranges of all measurements used for TvsL evaluation are given as median (10th; 90th percentile) and amount to OHI: 51 (28; 87), StO_2_: 28% (17%; 45%), NIR PI: 34 (12; 51), and TWI: 74 (57; 91).

Spearman’s rank correlation coefficients showed no significant correlation between MAE of tissue parameters and the time differences between the first HSI measurement and (a) switching on the light source, (b) the resection, or (c) the white balancing. The registration error of the corresponding fields of view and the mean parameter value of the record revealed no significant correlation with the MAE or the RMSE.

## Discussion

Compact hyperspectral imaging, suitable for routine laparoscopic use, enables tissue perfusion quantification and better tissue discrimination to support the visual perception during minimally invasive surgical procedures. The HSI MIS was designed for this purpose and successfully evaluated with *ex vivo* human tissue during this study.

Physiological tissue parameters of tumor resectates were calculated from hyperspectral data acquired with two HSI systems for open and laparoscopic use. As expected, the MAE between both systems (TvsL) was higher compared to different measurements with the same system (TvsT, LvsL) and two systems for open surgery (T1vsT2). Despite the rigorously controlled setup for high comparability of both systems, the MAE was nearly and over 10 units for all perfusion-related tissue parameters.

None of the investigated external influences due to the study design showed a significant correlation with the observed MAE of the tissue parameters or RMSE of the reflectance spectra. The latter was higher in the visible and lower in the near-infrared spectral range for the measurements TvsT and T1vsT2, whereas LvsL and TvsL showed the opposite.

This is in line with the comparison of the signal-to-noise ratio (SNR) between the TIVITA® Tissue and the predecessor of the HSI MIS reported previously by our group [28]. Moreover, the RMSE of the mean reflectance spectra measured with both systems on eight regions of a resectate were calculated in that study. The obtained average RMSE from 550 to 960 nm was 0.09, which complies with the results in the visible range presented here. Although the tissue parameters of both systems correspond qualitatively, it was shown that quantitative differences are not mainly caused by the study design, as previously expected [28]. Therefore, the main influences for deviations between both systems are probably the different light sources (halogen and LED) and objective lenses, which are not completely compensated by calibration.

An extended comparison of a halogen, xenon, and LED light source with regard to the influence on the SNR can be found in [18]. Although the halogen light source provides higher SNR values in the spectral range between 600 and 1000 nm as well as a more homogeneous lighting across the imaged area, the LED light source performs better from 500 to 600 nm. Because of further advantages, for example, higher lifetime, less heat development and therefore higher efficiency, LEDs will become the standard lighting unit in future. To reduce the above-mentioned deviations in physiological tissue parameters, algorithms and data processing methods are adapted and optimized in ongoing investigations.

This study is limited by the small number of patients and the nature of *ex vivo* investigations, in particular, the missing perfusion and reduced range of perfusion-related tissue parameters. It was not possible to investigate the deviations between two HSI MIS since only one system was available.

Nevertheless, this study contributes to making HSI useful for minimally invasive surgery in clinical practice since technical opportunities and limitations have been investigated in an *ex viv*o trial. Furthermore, the video shows the clinical readership future areas of application. To assess the comparability of the TIVITA® Tissue and HSI MIS during in vivo practical use, a clinical study with more patients has been initiated currently.

## Supplementary Information

Below is the link to the electronic supplementary material.

Supplementary file1 (MOV 1337663 kb)

Supplementary file2 (PART 1224180 kb)
